# CO-Induced TTP Activation Alleviates Cellular Senescence and Age-Dependent Hepatic Steatosis *via* Downregulation of *PAI-1*

**DOI:** 10.14336/AD.2023.0120

**Published:** 2023-04-01

**Authors:** Jeongmin Park, Yingqing Chen, Jeongha Kim, Eunyeong Hwang, Gyu Hwan Park, Chae Ha Yang, Stefan W Ryter, Jeong Woo Park, Hun Taeg Chung, Yeonsoo Joe

**Affiliations:** ^1^School of Biological Sciences, University of Ulsan, Ulsan, 44610, Korea.; ^2^Department of Pharmacology, Dalian University Medical College, Dalian, China.; ^3^College of Korean Medicine, Daegu Haany University, Daegu 42158, Korea.; ^4^College of Pharmacy, Research Institute of Pharmaceutical Sciences, Kyungpook National University, Daegu, Korea.; ^5^Proterris Inc., Boston, MA 02118, USA.

**Keywords:** aging, carbon monoxide, cellular senescence, non-alcoholic fatty liver diseases (NAFLD), stress granules, PAI-1, Sirt1, tristetraprolin

## Abstract

Aging can increase the risk of various hepatic diseases, especially non-alcoholic fatty liver disease (NAFLD). Although the mechanisms underlying the pathogenesis of age-related disorders such as NAFLD remain incompletely understood, recent studies have implicated the accumulation of senescent cells as a contributing factor. Here, we show that tristetraprolin (TTP) deficiency accelerates NAFLD during aging by enhancing the senescence-associated secretory phenotype (SASP) as well as several hallmarks of senescence. The sequestration of plasminogen activator inhibitor (PAI)-1, a mediator of cellular senescence, in stress granules, (SGs) inhibits cellular senescence. In our previous report, we have shown that carbon monoxide (CO), a small gaseous mediator, can induce the assembly of SGs *via* an integrated stress response. Here, we show that CO treatment promotes the assembly of SGs which can sequester PAI-1, resulting in the inhibition of etoposide (ETO)-induced cellular senescence. Notably, CO-induced TTP activation enhances PAI-1 degradation, leading to protection against ETO-induced cellular senescence. CO-dependent Sirt1 activation promotes the inclusion of TTP into SGs, leading to decreased PAI-1 levels. Therefore, our findings highlight the importance of TTP as a therapeutic target in age-related NAFLD and offer a potential new strategy to reduce the detrimental effects of senescent cells in hepatic disorders.

## INTRODUCTION

Aging is emerging as the major risk factor for chronic diseases such as neurodegenerative diseases [1, 2], cancer [3, 4], diabetes [5], and cardiovascular disease [6, 7]. Notably, aging has a significant impact on the severity of various hepatic diseases including non-alcoholic fatty liver disease (NAFLD), alcoholic liver disease, hepatitis C, and liver transplantation. Indeed, the human population shows an increased prevalence of NAFLD with age [8, 9]. The mechanisms underlying age-associated NAFLD are, however, not yet known.

Aging is promoted by cellular senescence which is a state of irreversible cell cycle arrest caused by a variety of stressors, including telomere shortening [10], DNA damage [11], epigenetic alteration [12], oxidative stress [13], and mitochondrial dysfunction [14]. Importantly, the accumulation of senescent cells during aging can contribute to aging and aging-related diseases. Several studies have reported that the elimination of senescent cells alleviates the symptoms of aging [15] and various age-related diseases [16]. Senescent cells typically appear flattened, enlarged, and show increased cytoplasmic granularity [17]. In addition, senescent cells also display several other characteristics that differ from proliferating cells. These differences include the increase of senescence-associated β-galactosidase (SA-β-gal) activity [18], increase of phosphorylated H2A histone family member X (γ-H2AX) foci [19], increased expression of cyclin-dependent kinase inhibitors (CDKIs) such as p21^CIP1^ and p16^INK4a^ [20, 21], as well as senescence associated secreted phenotype (SASP) which consists of growth hormones, pro-inflammatory cytokines, chemokines, angiogenic factors and extracellular matrix (ECM)-remodeling proteases [22, 23]. Recent studies suggest that the increased secretion of serine protease inhibitor, plasminogen activator inhibitor 1 (PAI-1), a component of SASP, can accelerate aging in mice. PAI-1 is a marker and critical mediator of cellular senescence [24]. Furthermore, senescence-inducing signals such as the DNA-damage response (DDR) and oxidative stress can enhance the activation of tumor suppressor p53, which triggers the expression and secretion of PAI-1. In turn, PAI-1 prevents cyclin D1-dependent phosphorylation of Rb, resulting in the irreversible cell cycle arrest [25]. Therefore, the inhibition of cellular senescence may be an attractive therapeutic target in age-related diseases.

In response to diverse environmental stresses, including heat, hyperosmolarity and oxidative stresses, eukaryotic cells temporarily cease protein synthesis to control energy expenditure for the repair of stress-induced damage. One of the major underlying mechanisms is the formation of stress granules (SG) in the cytoplasm. These non-membrane-bound SGs can arrest mRNAs and several harmful proteins to protect cells from apoptosis [26, 27]. SG biogenesis is recognized as a conserved stress response, which can be initiated by the oligomerization of Ras GTPase-activating protein-binding protein-1 (G3BP1) and aggregation of RNA binding proteins, including T-cell intracytoplasmic antigen (TIA-1), TIA1 related protein (TIAR) and HuR [28]. Notably, tristetraprolin (TTP), an AU-rich element (ARE)-containing mRNA binding protein, is excluded from SGs through activation of p38 mitogen activated protein kinase (p38 MAPK)/MAPK-activated protein kinase 2 (MK2) cascade [29]. Furthermore, SGs formation can inhibit cellular senescence *via* sequestration of PAI-1, and subsequently enhance the cyclin D1 pathway to remove cell cycle arrest [30].

Carbon monoxide (CO) is an endogenous gaseous mediator that is produced from heme by the activation of heme oxygenase-1 (HO-1), a stress-inducible response. When applied at low concentration, CO can exert cyto- and tissue- protective effects in various models of cellular and tissue injuries, involving anti-inflammatory, antioxidant, and anti-apoptotic effects [31]. Intriguingly, our recent study has demonstrated that CO can induce the formation of SGs through protein kinase RNA-like endoplasmic reticulum kinase (PERK)-eIF2α signaling pathway, a component of the integrated stress response (ISR) [32]. In addition, CO promotes the increase of TTP levels and its activation by regulation of phosphorylation and acetylation [33]. In this study, we found that CO promotes the sequestration of PAI-1 in SGs and CO-induced TTP activation enhances PAI-1 degradation in SG assembly. Finally, we suggest that TTP may present a new target molecule in age-related NAFLD.

## MATERIALS AND METHODS

### Reagents

CO-releasing molecule 2 (CORM2) and etoposide (ETO) were from Sigma-Aldrich (St Louis, MO, USA).

### Animals

TTP KO mice (*Ttp*^-/-^), in C57BL/6 background, were kindly provided by Dr. Perry J. Blackshear (Laboratory of Signal Transduction, National Institute of Environmental Health Sciences, USA). All mice were bred in the animal facility at the University of Ulsan and were born and housed under specific pathogen-free conditions at 18-24 ? and 40-70 % humidity, with a 12 h light-dark cycle. Animal studies were approved by the University of Ulsan Animal Care and Use Committee (Reference number HTC-19-020). To study liver aging, at the age of 10, 24, and 96 weeks, mice were anesthetized with intraperitoneal Avertin (250 mg/kg, Sigma-Aldrich), and liver tissues and serum from WT and *Ttp*^-/-^ male and female mice were collected for various assays.

### Cell culture

The human diploid cell line WI-38 and mouse liver cell line AML12 were cultured in Minimum Essential Medium (MEM, GIBCO, Grand Island, USA) and DMEM/F12 (GIBCO, Grand Island, USA), respectively, with 10% fetal bovine serum (FBS, GIBCO, Melbourne, Australia) and 1% penicillin-streptomycin (GIBCO) solution. Primary MEFs were isolated from E14.5 C57BL/6 embryos [34], the products of the mating of TTP heterozygous mice, and cultured in DMEM (GIBCO) medium with 10% FBS, 1% penicillin-streptomycin, and 1% MEM non-essential amino acid solution (GIBCO). The genotypes from each litter were determined by assessment of genomic DNA from each embryo. Primary hepatocytes were isolated from *Ttp*^-/-^ mice at the age of 24 and 96 weeks as previously described [35]. The liver tissues were perfused with Ca^2+^ and Mg^2+^-free Hanks’ buffered salt solution (HBSS, GIBCO), followed by perfusion with 0.2% collagenase type IV in Williams’ Medium E (GIBCO). The hepatocytes were cultured with DMEM (GIBCO) medium containing 10% FBS and 1% penicillin-streptomycin. Cells were grown at 37? in humidified incubators containing an atmosphere of 5% CO_2_.

### Transfection with siRNA

To knock down the mRNA expression of PAI-1 and TTP, cells were transfected with scramble siRNA (scRNA) (Ambion, Austin, TX, USA), used as negative control, siRNA against human PAI-1, mouse PAI-1, and mouse TTP (Santa Cruz Biotechnology, CA, USA) by applying Lipofectamine 2000 (Invitrogen, Carlsbad, CA, USA) according to the manufacturer’s protocol.

### RNA isolation and Reverse Transcription-Polymerase Chain Reaction

Total RNA was isolated from cells and liver tissues by utilizing QIAzol Lysis reagent (QIAGEN, Valencia, CA, USA), according to the manufacturer’s instructions. In brief, 2 μg of total RNA was used to generate cDNA using M-MLV reverse transcriptase (Promega, Madison, WI, USA). The synthesized cDNA was subject to PCR-based amplification. The following primers were used: mouse GAPDH (F-AGGCCGGTGCTGAGTATGTC, R-TGCC TGCTTCACCTTCT), mouse TTP (F-CTCTGCCATCT ACGAGAGCC, R-GATGGAGTCCGAGTTTATGTTC C), and mouse PAI-1 (F-GACGTTGTGGAACTGC CCTA, R-GACCTTTTGCAGTGCCTGTG). To perform quantitative real-time PCR (qRT-PCR), the synthesized cDNA was amplified with SYBR Green qPCR Master Mix (Applied Biosystems, Foster City, CA, USA) on ABI 7500 Fast Real-time PCR system (Applied Biosystems). The following primers were mouse GAPDH (F-GGGAAGCCCATCACCATCT, R-CGGCCTCACCCC ATTTG), mouse p21 (F-GTGGCCTTGTCGCTGT CTT, R-GCGCTTGGAGTGATAGAAATCTG), mouse p16 (F-AATCTCCGCGAGGAAAGC, R-GTCTGCAGCGG ACTCCAT), mouse PAI-1 (F-ACTGTCCTATCTCAA GGTCCACTGT, R-TGATCTGTCTATCCGTTG CCC), mouse TNF-α (F-AGACCCTCACACTCAGATCATC TTC, R-TTGCTACGACGTGGGCTACA), mouse IL-6 (F-CCAGAGATACAAAGAAATGATGG, R-ACTCC AGAAGACCAGAGGAAAT), mouse IL-1β (F-TCGC TCAGGGTCACAAGAAA, R-ATCAGAGGCAAGGA GGAAACAC), human GAPDH (F-CAATGACCCCTT CATTGACCTC, R-AGCATCGCCCCAC TTGATT), human p21 (F-CGATGGAACTTCGACTTT GTCA, R-GCACAAGGGTACAAGACAGTG), human PAI-1 (F-TGATGGCTCAGACCAACAAG, R-CAGCAATGAA CATGCTGAGG), human TNF-α (F-GCTGCACTTTG GAGTGATCG, R-GTTTGCTACAACATGGGCTACA G), human IL-6 (F-ACTCACCTCTTCAGAACGAATT G, R-CCATCTTTGGAAGGTTCAGGTTG) human IL-1β (F-TTACAGTGGCAATGAGGATGAC, R-GTCGG AGATTCGTAGCTGGAT).

### Western blotting

Lysates of cells and harvested liver tissues were prepared using RIPA buffer (Thermo Scientific, Waltham, MA, USA) containing protease inhibitor (Sigma-Aldrich), phosphatase inhibitor cocktail 2 (Sigma-Aldrich), and phosphatase inhibitor cocktail 3 (Sigma-Aldrich). Total protein concentration of the lysates was measured using a BCA protein assay kit (Pierce Biotechnology, Rockford, IL, USA). Proteins were resolved by SDS-PAGE, transferred onto polyvinylidene difluoride (PVDF) membranes (Millipore, Burlington, MA, USA), and probed with appropriate dilutions of the following antibodies: p53 (sc-6243, 1:1000, Santa Cruz), p21 (ab109199, 1:1000, Abcam, Cambridge, MA, USA), PAI-1 (sc-5297, 1:1000, Santa Cruz), TTP (T5327, 1:2000, Sigma-Aldrich), and α-tubulin (2125S, 1:1000, cell signaling, Danvers, MA, USA). Then, membranes were incubated with secondary antibodies (115-035-003; HRP-Goat Anti-Mouse IgG, 111-035-003; HRP-Goat Anti-Rabbit IgG) at room temperature for 30 min. Antibody binding was visualized with an ECL chemiluminescence system (Pierce Biotechnology) and chemiluminescence signal was read by Azure Biosystems C300 analyzer (Azure Biosystems, Dublin, CA). The relative band density was analyzed by using ImageJ2x software (US National Institutes of Health, Bethesda, USA).

### Enzyme-Linked Immunosorbent Assays (ELISA)

Cultured supernatant and mouse serum were collected, and the concentration of PAI-1 was measured by using a PAI-1 ELISA kit (BD Biosciences, San Jose, CA, USA), according to the manufacturer’s instructions. The concentration of pro-inflammatory cytokines, TNF-α, IL-6, and IL-1β, were analyzed in conditioned medium and measured by BioLegend ELISA kits (BioLegend, San Diego, CA, USA).

### Senescence-associated β-galactosidase staining

To observe the senescent cells, WI-38 and MEF cells were treated with etoposide to construct DNA damage induced premature cellular senescence. Then, senescence-associated (SA)-β-galactosidase (gal) staining was performed by utilizing a cellular senescence cell histochemical stain kit (Sigma-Aldrich) according to the manufacturer’s protocol. Briefly, after treatment, cells were washed with PBS and fixed with 4% paraformaldehyde, and SA-β-gal was stained by treatment with staining mixture. Five images of different sites per each well plate were obtained, and SA-β-gal-stained cells were counted. The percentage of senescent cells were analyzed by dividing the number of stained cells by the total number of cells.

### Immunofluorescence

Liver tissues, WI-38 cells, primary MEFs, and AML12 cells were plated on 4-well Lab-Tek chambered coverglass (Thermo Scientific, Waltham, MA, USA). After treatment, cells were washed in PBS, fixed with 4% (v/v) paraformaldehyde in PBS at room temperature for 15 min and permeabilized with 0.1% (v/v) Triton X-100 in PBS for 5min. Then, cells were washed three times with PBS and blocked with 3% BSA. To observe the formation of SGs and the sequestration of PAI-1, the samples were incubated with anti-TIA-1(ab40693, 1:500, Abcam), anti-G3BP1 (sc-365338, 1:200, Santa Cruz), anti-PAI-1 (sc-5297, 1:200, Santa Cruz) for 2 h at room temperature. To analyze senescence, cells were incubated with anti-γ-H2AX (05-636, 1:200, Millipore) for 2 h at room temperature. Cells were further washed three times with PBS before incubation for 1 h with Alexa Fluor 594 goat anti-rabbit IgG (A-11037, 1:500, Invitrogen) and Alexa Fluor 488 rabbit anti-mouse IgG (A-11059, 1:500, Invitrogen), respectively. Secondary antibodies were diluted in 1% (w/v) BSA in 1 x PBS. After incubation, cells were washed three times with PBS and stained with 1mg/ml DAPI (Sigma-Aldrich) for 15 min. Representative images were obtained using an Olympus FV1200 confocal microscope (Olympus, Tokyo, Japan). Rabbit IgG (ab172730, 1:500, Abcam) and mouse IgG1 (ab280974, 1:500, Abcam) were used as a negative control. The percentage of cells showing co-localization of SGs, PAI-1, and TTP, were determined. Images were analyzed for the number of cells with γ-H2AX foci. Each field contained at least 20 cells and three images per condition were analyzed.

### Luciferase activity

The 3’-UTR cDNA of human PAI-1 was PCR amplified using Taq polymerase (Bioneer, Daejeon, Korea). The following primers were used 5’-CCGCTCGAGC CCTGGGGAAAGACGCCTTCATCT-3’ AND 5’-AT TTGCGGCCGCGCTTCTATTAGATTACATTCATTT-3’. Plasmid psiCHECK2-PAI-1 3’UTR was generated by inserting the PCR product into the Xho-I and Not-I sites of the psiCHECK2 plasmid (Promega). To evaluate TTP-induced degradation of PAI-1 3’-UTR, cells were transfected with psiCHECK2-PAI-1 3’-UTR for 36 h and then treated with 20 and 40 μM CORM2 for 6 h. Luciferase activity was measured using a Dual-Luciferase Reporter Assay System (Promega) and a SpectraMax iD3 (Molecular Devices, Sunnyvale, CA, USA).

### Measurement of triglycerides

Hepatic triglycerides (TGs) were measured using a TG colorimetric assay kit (Cayman Chemical, Ann Arbor, MI, USA). Briefly, 50 mg samples of liver tissue were homogenized in 200 μl diluted standard diluents. After centrifugation, supernatants were obtained and were used for the assay.

### H&E staining

Liver tissues were fixed in 10% neutral-buffered formalin solution (Sigma-Aldrich) and sectioned with a cryostat at 5 μm. Tissue sections were mounted on regular glass slides and deparaffinized in xylene and rehydrated in graded alcohol series (anhydrous ethanol, 85% ethanol, 75% ethanol), then stained with hematoxylin for 3 min and eosin for 30 seconds.

### Hepatic damage assay

Activity of alanine aminotransferase (ALT) and aspartate aminotransferase (AST) in serum, as indicators of hepatic injury, were measured using the EnzyChrom ALT assay kit and EnzyChrom AST assay kit from BioAssay Systems (Hayward, CA, USA).

### Statistical analysis

All data were expressed as mean ± SD, which is representative of at least 3 independent experiments with a minimum of 3 biological replicates. The Shapiro-Wilk test was used to normality test of the data. Statistical significance between two groups were assessed by the Student’s t test (passed normality test) or the non-parametric Mann-Whitney U test (did not pass normality test or n < 6). To analyze the three or more groups, one-way analysis of variance (ANOVA) with repeated measures followed by Tukey *post hoc* test was performed for normally distributed data, and the Kruskal-Wallis test followed by the Dunn *post hoc* test was used to analyze non-normally distributed data. To analyze differences between WT mice and *Ttp*^-/-^ mice, data were evaluated by two-way ANOVA with Bonferroni post-tests. All statistical analysis were assessed by GraphPad Prism software version 9.3.1 (San Diego, CA, USA). The statistically significant changes among groups were considered as probability values of *p* ≤ 0.05.

**Figure 1.**
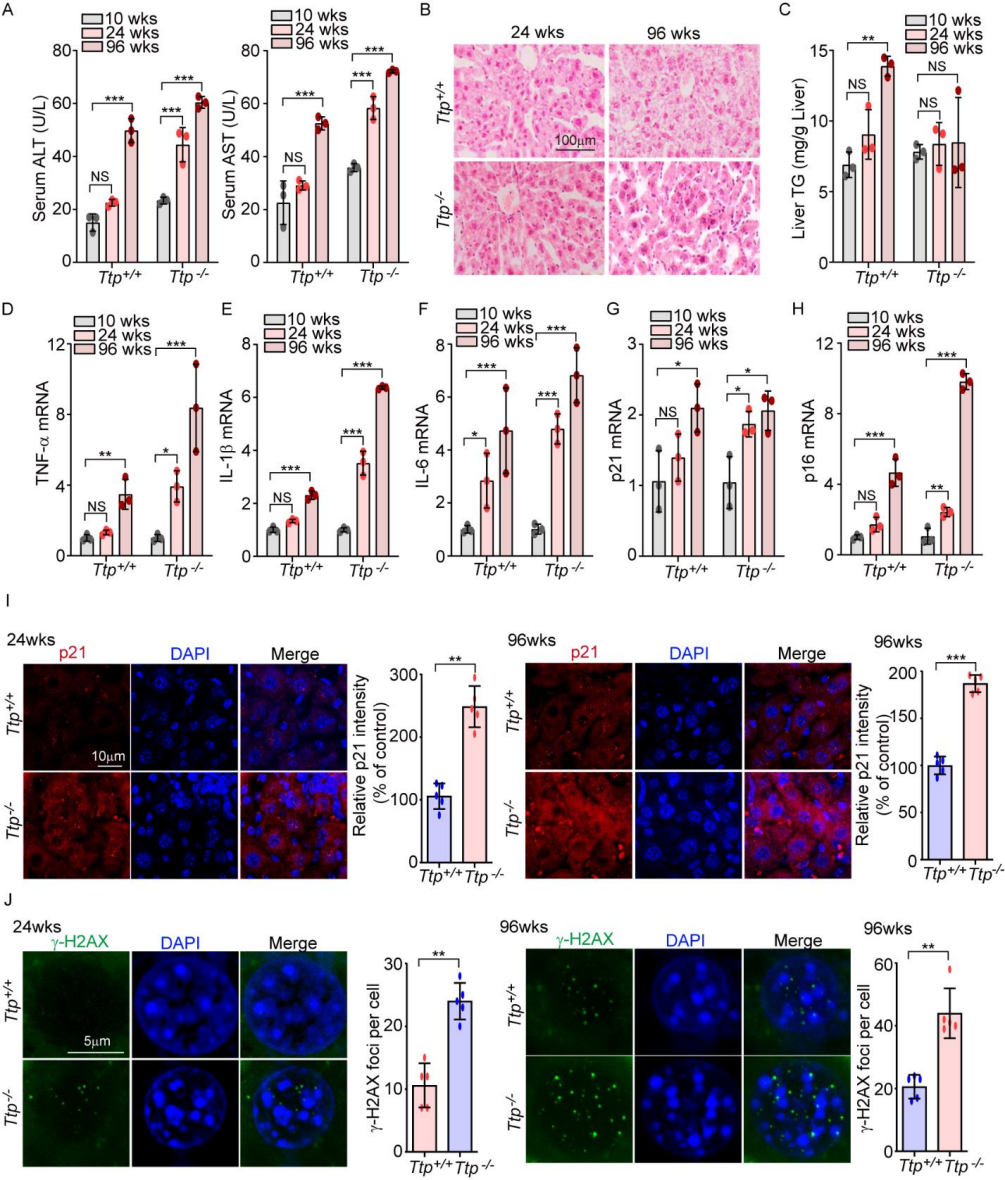
TTP attenuates aging-related hepatic dysfunction in mice. (A) Levels of serum ALT and AST were measured in WT (*Ttp*^+/+^) and TTP KO (*Ttp*^-/-^) mice (*n*=3 mice in each group) at the age of 10, 24, and 96 weeks. (B) Representative H&E-stained liver sections from *Ttp^+/+^* and *Ttp^-/-^* mice at 24 weeks and 96 weeks of age. Scale bar: 100 μm. (C) Levels of liver triglycerides were measured in *Ttp*^+/+^ mice and *Ttp*^-/-^ mice (*n*=3 mice in each group) at 10, 24, and 96 weeks of age. (D-H) The mRNA expression of senescence-associated secretory phenotype (SASP)-related genes, (D) TNF-α, (E) IL-1β, and (F) IL-6, and cellular senescence markers, (G) p21 and (H) p16, were assessed by qRT-PCR in liver tissues from *Ttp^+/+^* and *Ttp^-/-^* mice at the indicated ages. (A-H) Data were analyzed using the two-way ANOVA followed by Bonferroni post-test and expressed as the mean ± SD; ^*^p<0.05, ^**^p<0.01, ^***^p<0.001, and NS, not significant. (I) Liver sections at the age of 24 and 96 weeks were stained with anti-p21 antibody. Images of p21 immunofluorescence were detected by confocal microscopy, scale bar: 10 μm (*left*), and quantification of fluorescence intensity was analyzed (*right*). (J) γ-H2AX nuclear foci in liver sections were determined by immunofluorescence, scale bar: 5 μm (*left*). The number of γ-H2AX nuclear foci was counted (*right*). Rabbit IgG and mouse IgG1 were used as a negative control of anti-p21 antibody and anti-γ-H2AX antibody, respectively. (I, J) Data were analyzed using the Mann-Whitney U test and expressed as means ± SD; *n*=5 biological replicates; ^**^p<0.01 and ^***^p<0.001.

## RESULTS

### TTP attenuates aging-related hepatic dysfunction in mice

To explore the potential role of TTP in aging-related hepatic steatosis, we first measured serum ALT and AST levels as markers of liver damage in aging wild type (WT, *Ttp*^+/+^) or TTP knockout (KO, *Ttp*^-/-^) mice (Fig. 1A). The serum ALT and AST levels in middle-aged (24 weeks) or aged (96 weeks) *Ttp*^-/-^ mice were higher than in young (10 weeks) *Ttp*^-/-^ mice. There was no difference between the serum ALT and AST levels middle-aged (24 weeks) vs. young (10 weeks) WT mice. However, as expected, there was a marked increase of hepatic damage markers in extremely old mice (96 weeks), relative to young mice of both strains, with a trend toward higher levels in *Ttp*^-/-^ mice. We observed severe liver damage including steatosis and inflammation in the liver of aged *Ttp*^-/-^ mice relative to WT mice, using H&E staining (Fig. 1B). As shown in Fig. 1C, the levels of liver triglycerides (TGs) were increased in aged WT mice relative to young WT mice; but were not elevated in aged *Ttp*^-/-^ mice relative to younger *Ttp*^-/-^ mice. These results suggest that severe inflammation in aged *Ttp*^-/-^ mice may prevent an increase in liver TGs due to inflammation-induced hepatocyte cell death. In addition, we measured several SASPs, including the cytokines TNF-α, IL-1β, and IL-6, and the cell cycle regulators of p21 and p16. Old (96 weeks) *Ttp*^-/-^ mice displayed an increase in the levels of the SASPs; TNF-α, IL-1β, and IL-6 in the liver (Fig. 1D, 1E, and 1F) relative to younger *Ttp*^-/-^ mice, and higher values relative to old WT mice. In old mice, p21 and p16 were highly expressed in both WT and *Ttp*^-/-^ mice, with p16 displaying higher levels in the *Ttp*^-/-^ mice (Fig. 1G and 1H). To investigate the role of TTP in aging, we measured p21 and the number of γ-H2AX foci per cell in the livers of WT and *Ttp*^-/-^ mice. Both p21 staining (Fig. 1I) and the number of γ-H2AX foci (Fig. 1J) were significantly increased in the liver of *Ttp*^-/-^ mice compared to the liver of WT mice, in both middle-aged mice (24 weeks) (*left panels*) and aged mice (96 weeks) (*right panels*). Taken together, we suggest that TTP can prevent liver injury and hepatocyte cell senescence during aging.

### TTP deficiency facilitates age-dependent senescence via increasing PAI-1 expression in the liver

**Figure 2.**
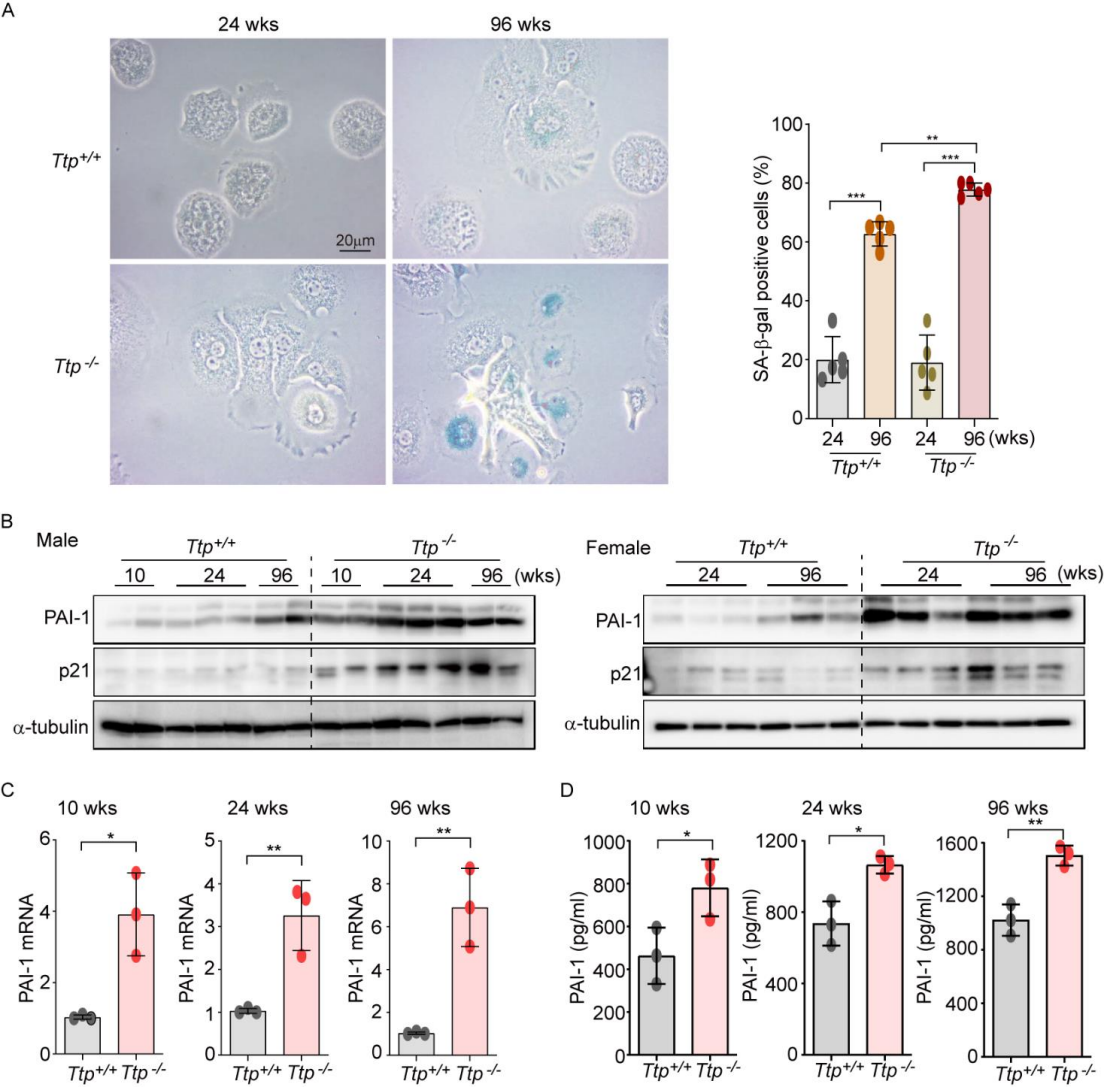
TTP deficiency facilitates age-dependent senescence *via* increasing PAI-1 expression in the liver. (A) Bright field microscopy images of SA-β-gal staining of primary hepatocyte isolated from WT (*Ttp*^+/+^) mice and TTP KO (*Ttp*^-/-^) mice at the age of 24 and 96 weeks (*left*). Cells were analyzed to calculate the percentage of SA-β-gal-positive cells (*right*). Data were analyzed using the two-way ANOVA followed by Bonferroni post-test and expressed as the mean ± SD; *n*=5 biological replicates; ^**^p<0.01 and ^***^p<0.001. (B) In liver tissues from WT and *Ttp^-/-^* male (*left*) and female (*right*) mice (*n*=3 mice in each group), the expression levels of PAI-1 and p21 were determined by immunoblot analysis at the indicated ages. (C, D) The expression levels of (C) hepatic mRNA and (D) serum levels of PAI-1 were analyzed by qRT-PCR and ELISA, respectively, in *Ttp^+/+^* and *Ttp^-/-^* mice at the ages of 10 (*left*), 24 (*middle*), and 96 (*right*) weeks. Data were analyzed using the Mann-Whitney U test and expressed as means ± SD; *n*=3 mice in each group; ^*^p<0.05 and ^**^p<0.01.

To investigate whether TTP is involved in aging and senescence, primary hepatocytes from WT and *Ttp*^-/-^ mice were stained with SA-β-gal. The percentage of SA-β-gal positive cells significantly increased in aged mice of both strains (96 weeks) compared to middle-aged mice (24 weeks). Aged *Ttp*^-/-^ mice (96 weeks) displayed more SA-β-gal positive cells compared to aged WT mice (Fig. 2A). Given that the expression of the PAI-1 gene is markedly stimulated in various aging-associated pathologies [36], we investigated whether PAI-1 levels are regulated during aging in a TTP-dependent manner. The levels of PAI-1 protein were elevated with increased age in WT mice; PAI-1 protein levels were increased in *Ttp*^-/-^ mice relative to WT mice in all age groups (Fig. 2B). We also measured the levels of the senescence marker p21 to determine whether an increase of PAI-1 in *Ttp*^-/-^ mice was associated with changes in p21 status. The levels of p21 were increased in an aged-dependent manner in *Ttp*^-/-^ mice (Fig. 2B). Consistent with results observed in male mice, age-dependent increases of PAI-1 and p21 levels were also observed in female *Ttp*^-/-^ mice (Fig. 2B). In addition, TTP deficiency was associated with an increase in mRNA expression of PAI-1 in all age groups (Fig. 2C). Secreted PAI-1 levels were also increased in *Ttp*^-/-^ mice compared to WT mice (Fig. 2D). Therefore, TTP may prevent age-associated senescence phenotypes *via* decreasing PAI-1 levels.

### CO inhibits etoposide-induced cellular senescence in human and murine fibroblasts

Several studies have reported that topoisomerase inhibitors, such as ETO, doxorubicin and topotecan, which are commonly used as chemotherapeutic agents, can induce DNA double strand breaks (DSBs) in tumor cells, and these lesions can be toxic to normal cells [37]. In addition, these drugs are reported as potent inducers of premature senescence in normal human fibroblasts *via* activating p53 [38].

**Figure 3.**
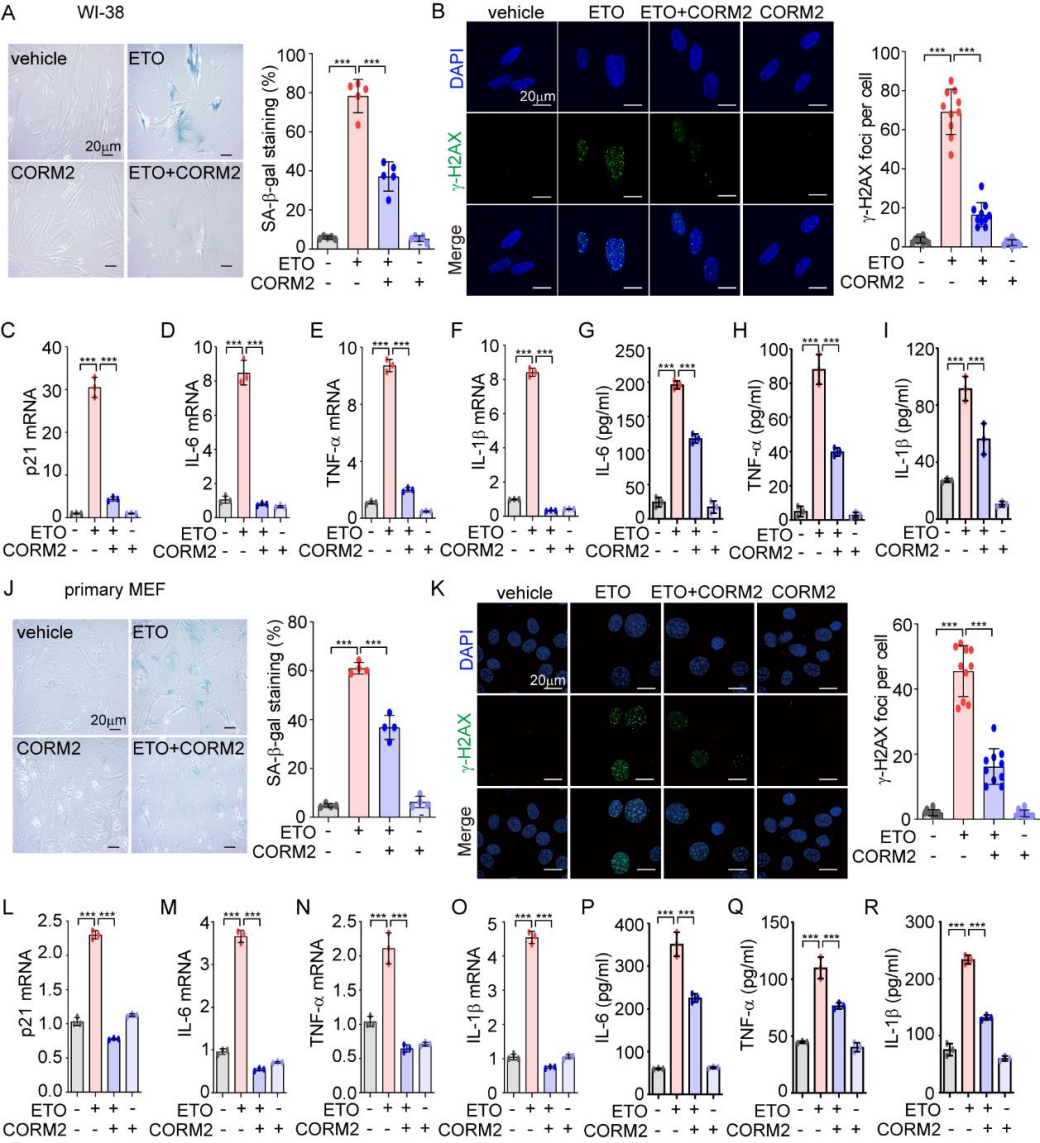
CO inhibits ETO-induced cellular senescence in human and murine fibroblasts. (A-I) WI-38 cells were pretreated with CORM2 (40 μM) for 6 h followed by stimulation with ETO (80 μM) for 24 h, and then cells were changed into fresh media. During the process of senescence, cells were treated with CORM2 (40 μM) for 6 h every two days, and after 7-days incubation, SA-β-gal staining was performed. (A) Representative images of SA-β-gal staining (*left*), scale bar: 20 μm. Quantification of the SA-β-gal positive cells is shown in the right panel (mean ± SD; *n*=5 biological replicates; ^***^p<0.001; Kruskal-Wallis test followed by the Dunn *post hoc* test). (B) Immunofluorescence for detecting γ-H2AX foci was performed (mean ± SD; *n*=10 biological replicates; ^***^p<0.001; one-way ANOVA followed by Tukey *post hoc* test; Scale bar: 20 μm). Mouse IgG1 was used as a negative control of anti-γ-H2AX antibody. The mRNA expression levels of (C) p21, (D) IL-6, (E) TNF-α and (F) IL-1β were measured by qRT-PCR. The secretion levels of (G) IL-6, (H) TNF-α, and (I) IL-1β were measured by ELISA in cell culture supernatants. (C-I) Data were analyzed using Kruskal-Wallis test followed by the Dunn post hoc test and expressed as means ± SD; *n*=5 biological replicates; ^***^p<0.001. (J-R) Primary MEFs were pretreated with CORM2 (40 μM) for 6 h, and then cells were treated with ETO (2 μM) for 4 days. During the process of senescence, MEFs were treated with CORM2 (40 μM) for 6 h every two days. After 4-days incubation, cells were subjected with (J) SA-β-gal staining (mean ± SD; *n*=4 biological replicates; ^***^p<0.001; Kruskal-Wallis test followed by the Dunn post hoc test; Scale bar: 20 μm) and (K) stained with anti-γ-H2AX antibody for assessing γ-H2AX foci (mean ± SD; *n*=10 biological replicates; ^***^p<0.001; one-way ANOVA followed by Tukey post hoc test; Scale bar: 20 μm). Mouse IgG1 was used as a negative control of anti-γ-H2AX antibody. (L-O) The mRNA expression of (L) p21, (M) IL-6, (N) TNF-α, and (O) IL-1β were evaluated by qRT-PCR. (P-R) The levels of secreted (P) IL-6, (Q) TNF-α, and (R) IL-1β were detected by ELISA. (L-R) Data were analyzed using Kruskal-Wallis test followed by the Dunn *post hoc* test and expressed as means ± SD; *n*=3 biological replicates; ^***^p<0.001.

**Figure 4.**
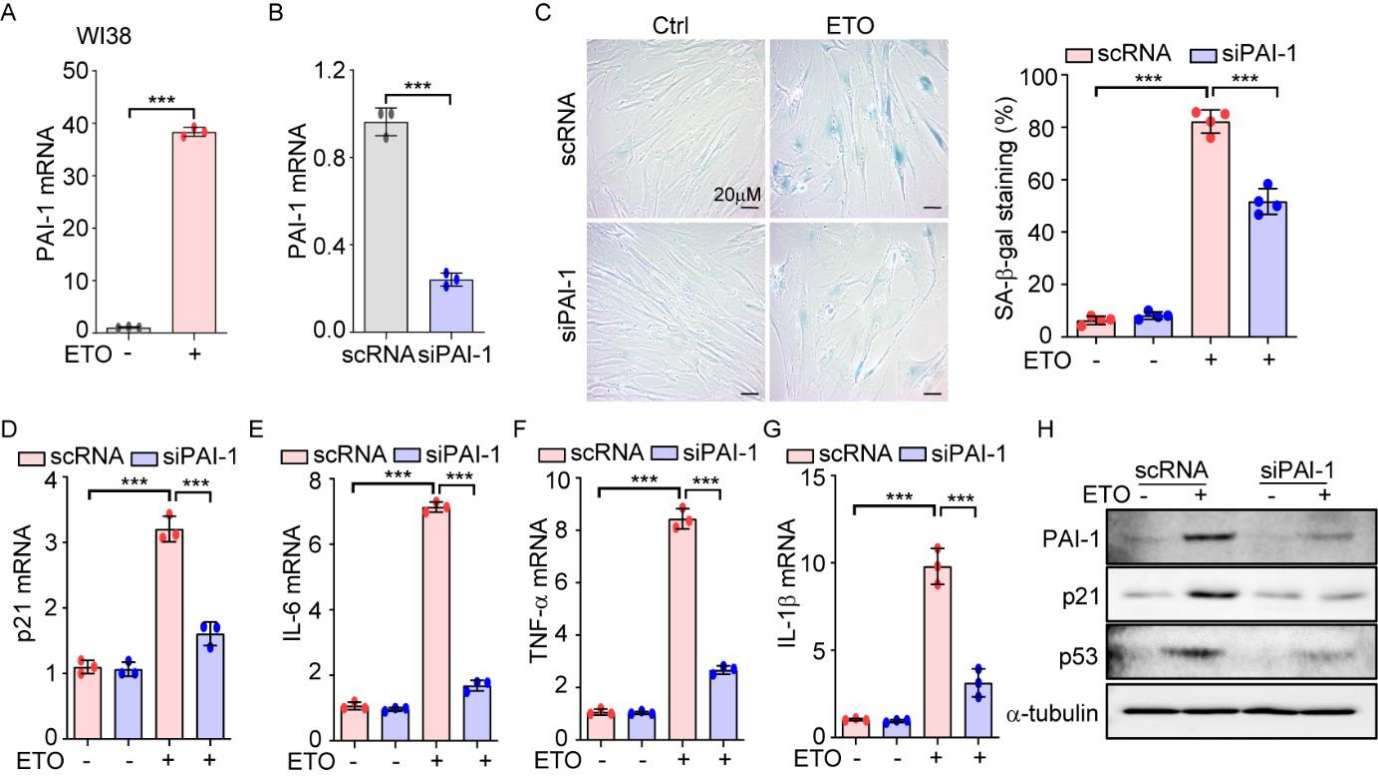
PAI-1 is involved in ETO-induced premature senescence in WI-38 and MEF cells. (A) WI-38 cells were treated with ETO (80 μM) for 24 h and then cells were refed with fresh media. After 7-day incubation, cells were harvested and the mRNA expression of PAI-1 was measured by qRT-PCR (mean ± SD; *n*=3 biological replicates; ^***^p<0.001; Mann-Whitney U test). (B-H) WI-38 cells were transfected with scramble siRNA (scRNA) and siRNA against PAI-1 (siPAI-1) for 36 h and then (B) PAI-1 mRNA level was assessed by qRT-PCR (mean ± SD; *n*=3 biological replicates; ^***^p<0.001; Mann-Whitney U test). Transfected cells were treated with ETO (80 μM) for 24 h and then cells were refed with fresh media. (C) After 7-day incubation, cells were subjected to SA-β-gal staining. Quantification of SA-β-gal-positive cells was shown in the right panel (mean ± SD; *n*=4 biological replicates; ^***^p<0.001; Kruskal-Wallis test followed by the Dunn *post hoc* test; Scale bar: 20 μm). (D-G) The mRNA expression of (D) p21, (E) IL-6, (F) TNF-α and (G) IL-1β were measured by qRT-PCR. Data were analyzed using Kruskal-Wallis test followed by the Dunn *post hoc* test and expressed as means ± SD; *n*=3 biological replicates; ^***^p<0.001. (H) The levels of protein expression of PAI-1, p21, and p53 were assessed by immunoblotting.

We investigated whether low dose CO exhibits inhibitory effects on the pro-senescence effects of ETO. We pre-treated the human fibroblast like fetal lung cell line, WI-38, with CORM, a CO-releasing molecule 2. At various concentrations (0, 10, 20, and 40 μM) for 6 h followed by the administration of ETO (80 μM) for 24 h. Then, the cells were cultured in fresh media, and were post-treated with CORM2 for 6 h every two days. After 7-days of incubation, we found that ETO alone significantly increased the number of cells positive for the expression of p21 and several SASPs, including IL-6, TNF-α, and IL-1β. However, treatment of WI-38 cells with low doses of CORM2 (20 and 40 μM) significantly reduced ETO-stimulated levels of p21 and SASPs, indicating that CORM2 may exert an anti-senescent effect on ETO-induced premature senescence (Supplementary Fig. 1A-1D). Given that 40 μM CORM2 was the optimal dose to reduce the levels of p21 and various SASPs, in the following studies, we chose 40 μM CORM2 as the appropriate dose to treat cells. To further assess the anti-senescent effect of CO, we treated WI-38 cells (Fig. 3A-3I) and primary mouse embryonic fibroblasts (MEFs) (Fig. 3J-3R) with or without CORM2 prior to the administration of ETO, and found that the enhanced markers of cellular senescence, including the percentage of senescence associated (SA)-β-gal positive cells (Fig. 3A and 3J), γ-H2AX foci (Fig. 3B and 3K), mRNA levels of p21 (Fig. 3C and 3L), IL-6 (Fig. 3D and 3M), TNF-α (Fig. 3E and 3N), and IL-1β (Fig. 3F and 3O); and secreted protein levels of IL-6 (Fig. 3G and 3P), TNF-α (Fig. 3H and 3Q), and IL-1β (Fig. 3I and 3R), were all significantly decreased by treatment with CORM2. To investigate whether exogenous CO gas can also protect against cellular senescence, we exposed cells to 250 ppm CO for 6 h every two days in the presence or absence of ETO. CO gas dramatically inhibited the corresponding senescence markers increased by ETO treatment (Supplementary Fig. 1E-1J). These results strongly suggested that CO can effectively prevent ETO-induced premature senescence.

**Figure 5.**
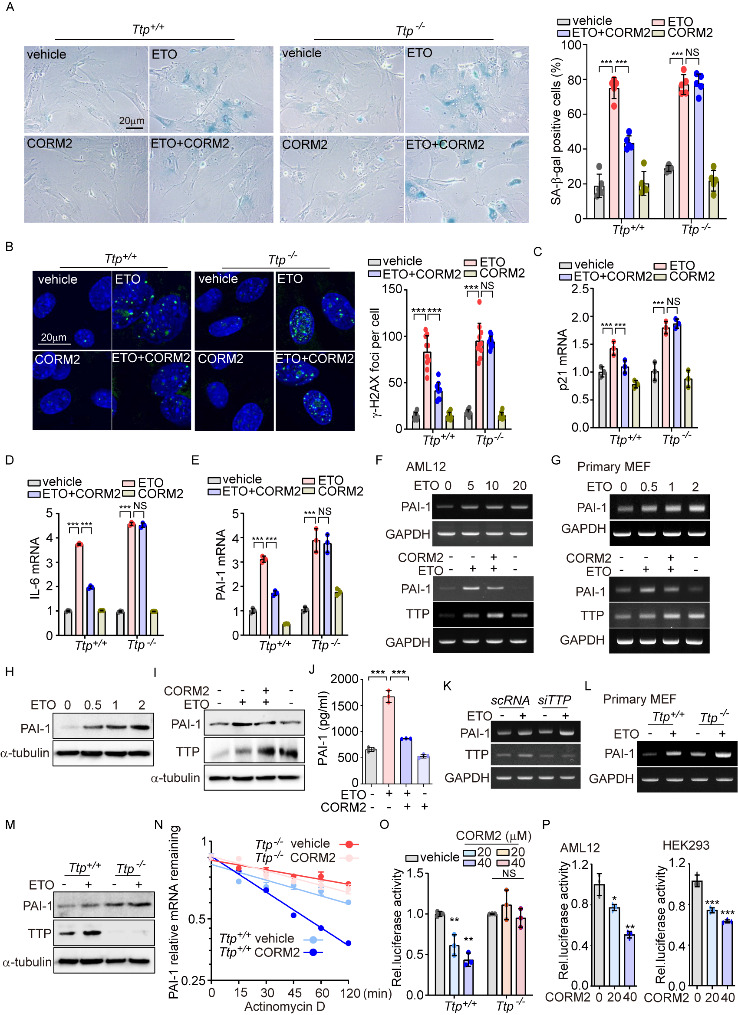
TTP is required for the inhibition of senescence by CO through PAI-1 downregulation. (A-E) WT (*Ttp*^+/+^) and TTP KO (*Ttp*^-/-^) primary MEFs were pretreated with CORM2 (40 μM) for 6 h and then the cells were treated with ETO (2 μM) for 4 days. During the process of senescence, MEFs were treated with CORM2 (40 μM) for 6 h every two days. (A) After 4 days of incubation, cells were stained with SA-β-gal. Scale bar: 20 μm (*left*). Cells were analyzed to calculate the percentage of SA-β-gal-positive cells (*right*; mean ± SD; *n*=5 biological replicates; ^***^p<0.001 and NS, not significant; two-way ANOVA followed by Bonferroni post-test). (B) Cells were performed with immunofluorescence for detecting γ-H2AX foci. Scale bar: 20 μm (*left*). Mouse IgG1 was used as negative control of anti-γ-H2AX antibody. The number of γ-H2AX nuclear foci was counted (*right*; mean ± SD; *n*=10 biological replicates; ^***^p<0.001 and NS, not significant; two-way ANOVA followed by Bonferroni post-test). The levels of mRNA expression of (C) p21, (D) IL-6, and (E) PAI-1 were detected by qRT-PCR. (C-E) Data were analyzed using the two-way ANOVA followed by Bonferroni post-test and expressed as the mean ± SD; *n*=3 biological replicates; ^***^p<0.001 and NS, not significant. (F) mRNA expression of PAI-1 and TTP in AML12 cells treatment with ETO (0, 5, 10, and 20 μM) for 4 days (*upper panel*) or treatment with 20 μM ETO in the presence or absence 40 μM CORM2 (*lower panel*). (G) mRNA levels of PAI-1 and TTP in primary MEFs treatment with ETO (0, 0.5, 1, and 2 μM) for 4 days (*upper panel*) or treatment with 40 μM CORM2 for 6 h followed by 20 μM ETO treatment (*lower panel*). (H) The levels of protein expression of PAI-1 in primary MEFs treatment with ETO (0, 0.5, 1, and 2 μM) for 4 days. (I) Protein levels of PAI-1 and TTP in pretreated with 40 μM CORM2 for 6 h, followed by treatment with 2 μM ETO for 4 days. (J) Secretion levels of PAI-1 in WI-38 cells were measured by ELISA in the indicated groups (mean ± SD; *n*=3 biological replicates; ^***^p<0.001; Kruskal-Wallis test followed by the Dunn post-hoc test). (K) AML12 cells were transfected with scramble siRNA (scRNA) and siRNA against TTP (siTTP) for 36 h and then treated with ETO (20 μM) for 4 days. mRNA levels of TTP and PAI-1 were assessed by RT-PCR. (L, M) The mRNA levels of PAI-1 (L) and protein levels of PAI-1 and TTP (M) in *Ttp^+/+^* and *Ttp^-/-^* primary MEFs treated with 2 μM ETO for 4 days. (N) Stability of PAI-1 expression at the indicated time points after actinomycin D (5 μg/ml) in *Ttp^+/+^* and *Ttp^-/-^* primary MEFs treated with 40 μM CORM2 for 6 h. (O, P) Luciferase activity in (O) *Ttp^+/+^* and *Ttp^-/-^* primary MEFs, (P) AML12, and HEK293 cells transfected with a psi-CHECK2-PAI-1 3’-UTR construct, followed by treatment with CORM2 (20 and 40 μM) for 6 h. (O) Data were analyzed using the two-way ANOVA followed by Bonferroni post-test and expressed as the mean ± SD; *n*=3 biological replicates; ^**^p<0.01 and NS, not significant. (P) Data were analyzed using Kruskal-Wallis test followed by the Dunn post hoc test and expressed as means ± SD; *n*=3 biological replicates; ^*^p<0.05, ^**^p<0.01, and ^***^p<0.001.

### PAI-1 mediates ETO-induced premature senescence in WI-38 and MEF cells

PAI-1 is a primary inhibitor of tissue type and urokinase type plasminogen activators, which convert plasminogen into plasmin, a serine proteinase that plays a major role in fibrinolysis [39]. Besides inhibition of fibrinolysis, several lines of evidence suggest that PAI-1 expression is increased in senescent cells and that PAI-1 is not only a marker but also a key mediator of cellular senescence and organismal aging [24]. To determine whether PAI-1 is increased in ETO-treated cells, we first assessed the mRNA expression of PAI-1 in WI-38 cells and primary MEFs. Our results showed that the expression of PAI-1 was significantly increased in both cell types after the administration of ETO (Fig. 4A and Supplementary Fig. 2A). Next, to evaluate whether increased PAI-1 expression is responsible for ETO-induced premature senescence, we transfected cells with siRNA against PAI-1 for 36 h (Fig. 4B and Supplementary Fig. 2B), and then the cells were stimulated with ETO. Silencing PAI-1 dramatically decreased markers of cellular senescence, including the percentage of SA-β-gal-stained cells (Fig. 4C and Fig. 2C), and the mRNA expressions of p21 (Fig. 4D and Supplementary Fig. 2D), IL-6 (Fig. 4E and Supplementary Fig. 2E), TNF-α (Fig. 4F and Supplementary Fig. 2F), and IL-1β ?Fig. 4G and Supplementary Fig. 2G) compared to cells transfected with scramble RNA. PAI-1 depletion abolished the ETO-induced increase in the protein levels of p21 and p53 in WI38 cells (Fig. 4H) and primary MEF cells (Supplementary Fig. 2H). These results suggest that ETO-induced cellular senescence is regulated by PAI-1 levels.

### TTP is required for inhibition of senescence by CO via downregulation of PAI-1

We demonstrated that TTP exerts a critical role in the protection against aging-dependent phenotypes *in vivo* (Fig. 1) and that TTP depletion increased PAI-1 levels (Fig. 2). In addition, CO generated from CORM2 treatment recovered ETO-induced cellular senescence and inhibited ETO-induced SASPs secretion (Fig. 3).

To find the underlying mechanisms by which TTP can regulate age-dependent processes, we analyzed the effects of CORM2 on ETO-induced senescence using primary MEFs isolated from WT and *Ttp*^-/-^ mice. CORM2 treatment suppressed ETO-induced SA-β gal positive cells in WT murine primary MEFs, but not in *Ttp*^-/-^ MEFs (Fig. 5A). In addition, cellular senescent phenotypes such as γ-H2AX foci (Fig. 5B), p21 mRNA levels (Fig. 5C), IL-6 mRNA levels (Fig. 5D), and PAI-1 mRNA levels (Fig. 5E) were increased by ETO in both WT murine and *Ttp*^-/-^ murine primary MEF. CORM2 treatment suppressed the cellular senescent phenotype in WT, but not in *Ttp*^-/-^ MEFs (Fig. 5B-5E). We also confirmed that ETO increased PAI-1 mRNA levels in a dose-dependent manner in AML-12 cells (Fig. 5F) and in primary MEFs (Fig. 5G). CORM2 treatment inhibited ETO-induced PAI-1 mRNA levels in AML-12 cells (Fig. 5F) and in primary MEFs (Fig. 5G). Also, the levels of PAI-1 protein were increased by ETO treatment in primary MEFs in a dose-dependent manner (Fig. 5H). In addition, we confirmed that the increases in PAI-1 protein levels (Fig. 5I) and PAI-1 secretion (Fig. 5J) by ETO were suppressed by CORM2 treatment. TTP knock-down using siRNA against TTP resulted in increased expression of PAI-1 in response to ETO relative to cells transfected with scrambled (control) siRNA (Fig. 5K). ETO-induced PAI-1 mRNA and protein levels were enhanced in *Ttp*^-/-^ MEFs relative to WT MEFs (Fig. 5L and 5M).

**Figure 6.**
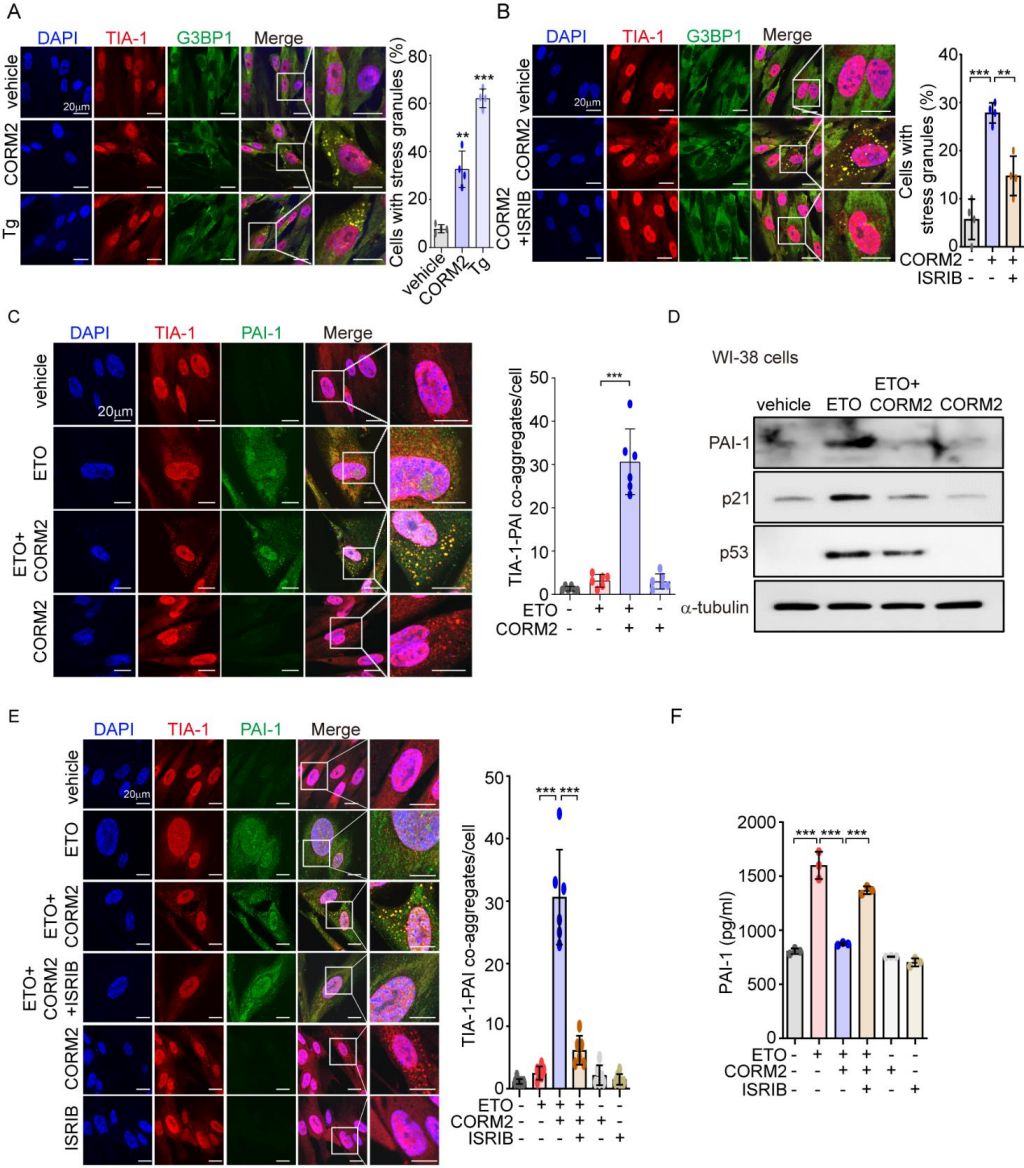
CO-induced SGs participate in reducing ETO-induced senescence by sequestration of PAI-1. (A) WI-38 cells were treated with 40 μM CORM2 for 6 h. As a positive control, WI-38 cells were treated with 200 nM thapsigargin (Tg) for 45 min and then an immunofluorescence assay was performed to detect the formation of SGs by visualizing the co-localization of TIA-1(red) and G3BP1 (green). Rabbit IgG and mouse IgG1 were used as a negative control of anti-TIA-1 antibody and anti-G3BP1 antibody, respectively. Scale bar: 20 μm (*left*). The percentage of cells containing SGs was analyzed and is shown in the right panel. (B) WI-38 cells were treated with 40 μM CORM2 in the presence or absence of ISRIB (200 nM) for 6 h and then stained with anti-TIA-1 and anti-G3BP1 antibodies. The formation of SGs was detected by visualizing the co-localization of TIA-1(red) and G3BP1 (green). Rabbit IgG and mouse IgG1 were used as negative control of anti-TIA-1 antibody and anti-G3BP1 antibody, respectively. Scale bar: 20 μm (*left*). Quantification is shown in the bar graphs on the right panel. (A, B) Data were analyzed using Kruskal-Wallis test followed by the Dunn *post hoc* test and expressed as means ± SD; *n*=3 biological replicates; ^**^p<0.01 and ^***^p<0.001. (C, D) WI-38 cells were pretreated with 40 μM CORM2 for 6 h followed by the administration of ETO (80μM) for 24 h and then cells were refed with fresh media. During the process of senescence, cells were post-treated with 40 μM CORM2 for 6 h. (C) After 7 days incubation, an immunofluorescence assay was performed to detect TIA-1 (red) and PAI-1 (green) co-aggregates. Rabbit IgG and mouse IgG1 were used as a negative control of anti-TIA-1 antibody and anti-PAI-1 antibody, respectively. Scale bar: 20 μm. The co-localization of TIA-1 and PAI-1 was quantified (*right*; mean ± SD; *n*=6 biological replicates; ^***^p<0.001; one-way ANOVA followed by Tukey *post hoc* test). (D) The levels of protein expression of PAI-1, p21, and p53 were detected by immunoblotting. (E, F) WI-38 cells were pretreated with CORM (40 μM) and ISRIB (200 nM) for 6 h followed by treatment with ETO (80 μM) for 24 h, and then cells were refed with fresh media. During the process of senescence, cells were post-treated with CORM2 (40 μM) and ISRIB (200 nM) for 6 h. (E) After 7 days incubation, cells were fixed to perform an immunofluorescence assay of PAI-1 and TIA-1 (*left*). Rabbit IgG and mouse IgG1 were used as a negative control of anti-TIA-1 antibody and anti-PAI-1 antibody, respectively. Scale bar: 20 μm. Quantification of co-localization of PAI-1 and TIA-1 is shown in the bar graphs to the right panel (mean ± SD; *n*=6 biological replicates; ^***^p<0.001; one-way ANOVA followed by Tukey *post hoc* test). (F) The secretion of PAI-1 was measured by ELISA (mean ± SD; *n*=3 biological replicates; ^***^p<0.001; Kruskal-Wallis test followed by the Dunn *post hoc* test).

To further investigate the effect of TTP on PAI-1 mRNA stabilization, we assayed PAI-1 mRNA stability with the actinomycin D assay, in WT and *Ttp*^-/-^ MEFs treated with CORM2. In WT MEFs treated with CORM2, PAI-1 mRNA degradation was accelerated compared to WT MEFs without CORM2 treatment and compared to *Ttp*^-/-^ MEFs (Fig. 5N). In addition, CORM2 reduced PAI-1 3’-UTR stability in WT, but not *Ttp*^-/-^ MEFs, as detected using a luciferase-based assay (Fig. 5O). Further, CORM2 treatment does-dependently lowered the PAI-1 3’-UTR stability in AML-12 and HEK293 cells (Fig. 5P). Therefore, CORM2-derived CO reversed ETO-induced cellular senescence *via* PAI-1 degradation, in cells expressing TTP.

### CO-induced SGs reduce ETO-induced senescence by sequestration of PAI-1

Assembly of SGs induced by constitutive stress can decrease the number of senescent cells through the recruitment of PAI-1 [30]. We reported that CO can stimulate the formation of SGs by selective induction of the PERK-eIF2α signaling pathway, a component of the integrated stress response (ISR) [32]. Based on these reports, we hypothesized that the anti-senescence effect of CO is mediated by assembly of SGs. Here, we first confirmed the beneficial effect of CO on SG formation by treating WI-38 cells with CORM2 or exogenous CO gas. Consistent with our earlier work [32], CORM2 (Fig. 6A) and CO gas (Supplementary Fig. 3A) significantly increased the assembly of TIA-1 and G3BP-1 positive SGs in cytoplasm. In addition, we confirmed that an ISR inhibitor (ISRIB) can markedly decrease the formation of SGs in response to CORM2 treatment (Fig. 6B). We also investigated whether CO could stimulate the sequestration of PAI-1 into SGs in ETO-treated WI-38 cells. Notably, the increased number of SGs initiated by CORM2 (Fig. 6C) and CO gas (Supplementary Fig. 3B) treatment significantly sequestrated PAI-1, as detected by co-aggregation of TIA-1 and PAI-1. In addition, ETO-induced PAI-1 protein levels were decreased by CORM2 in WI-38 cells (Fig. 6D). The levels of p21 and p53, as senescence-related proteins, were also enhanced by ETO; and these increases were inhibited by CORM2 (Fig. 6D and Supplementary Fig. 3E). Furthermore, we also observed that ISRIB strongly inhibits the sequestration of PAI-1 into CO-induced SGs (Fig. 6E), and consequently, the ability of CORM2 to decrease the secretion of PAI-1was abolished (Fig. 6F). As expected, co-treatment with ISRIB reversed the protective effects of CORM2 on ETO-induced senescence as measured by activity of SA-β-gal (Supplementary Fig. 3C), γ-H2AX foci (Supplementary Fig. 3D), and the protein levels of PAI-1, p21, and p53 (Supplementary Fig. 3E). Under the same conditions, we also measured the mRNA expression of p21 and several SASPs, such as IL-6, TNF-α, and IL-1β ? Supplementary Fig. 3F-3I ?. Together, these results suggest that CO-induced SGs prevent cellular senescence by sequestering PAI-1.

### CO-mediated inhibition of PAI-1 requires Sirt1-TTP activation and the assembly of SGs

**Figure 7.**
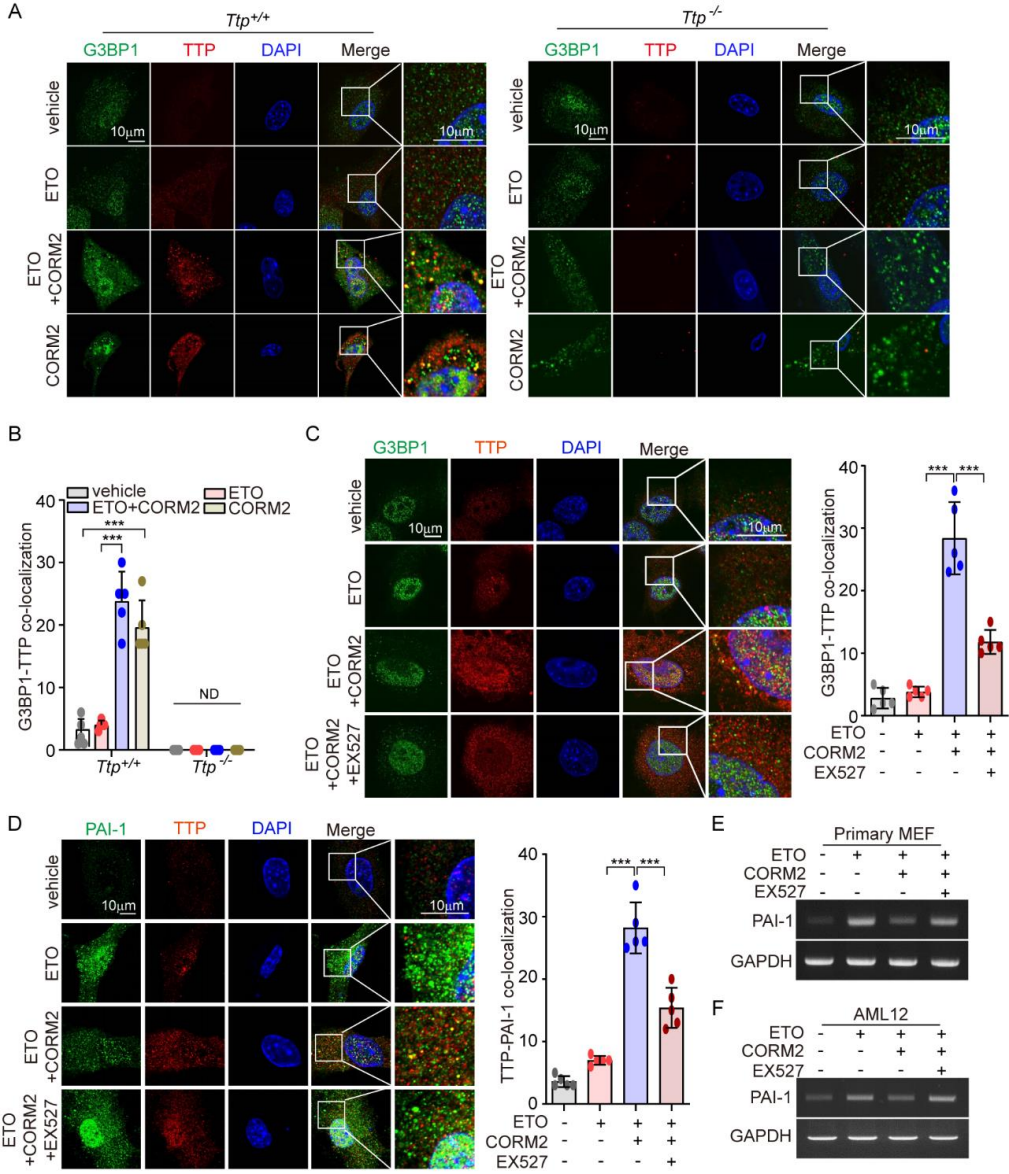
The decrease of PAI-1 by CO requires Sirt1-TTP activation in the assembly of SGs. (A, B) *Ttp^+/+^* and *Ttp^-/-^* primary MEFs were pretreated with CORM2 (40 μM) for 6 h and then cells were treated with ETO (2 μM) for 4 days. During the process of senescence, MEFs were treated with CORM2 (40 μM) for 6 h every two days. (A) After 4 days incubation, cells were stained with anti-TTP and anti-G3BP1 antibodies for assessing co-localization of SGs and TTP. Rabbit IgG and mouse IgG1 were used as a negative control of anti-TTP and anti-G3BP1 antibody, respectively. Scale bar: 10 μm. (B) Quantification of co-localization of G3BP1 and TTP is shown in the bar graphs (mean ± SD; *n*=5 biological replicates; ^***^p<0.001 and ND, not determined; two-way ANOVA followed by Bonferroni post-test). (C) AML12 cells were treated with 20 μM ETO for 4 days in the presence or absence of 40 μM CORM2 and 10 μM EX527 and cells were stained with anti-TTP and anti-G3BP1 antibodies (*left*). Rabbit IgG and mouse IgG1 were used as negative control of anti-TTP and anti-G3BP1 antibody, respectively. (D) Primary MEFs were treated with 20μM ETO for 4 days in the presence or absence of 40μM CORM2 and 10μM EX527 and cell were stained with anti-TTP and anti-PAI-1 antibodies (l*eft*). Rabbit IgG and mouse IgG1 were used as negative control of anti-TTP and anti-PAI-1 antibody, respectively. Quantification of co-localization of G3BP1 and TTP is shown in the right panel (mean ± SD; *n*=5 biological replicates; ^***^p<0.001; Kruskal-Wallis test followed by the Dunn *post hoc* test). (E, F) RT-PCR in (E) primary MEFs and (F) AML12 cells was performed to detect PAI-1 in the indicated groups.

SG-associated proteins such as TIA-1, TIAR, and HuR bind to ARE-containing mRNAs and control their translation and stability [40]. Under energy deprivation, TTP is also recruited to SGs, which contributes to degrading ARE-containing transcripts [40]. We demonstrated that CORM2 diminished ETO-induced PAI-1 expression in a TTP-dependent manner (Fig. 5) and promoted PAI-1 sequestration in SGs (Fig. 6). Thus, we investigated whether CORM2 can promote the recruitment of TTP to SGs. CORM2 increased TTP migration into SGs, leading to co-localization of G3BP1 and TTP in WT MEFs but not in *Ttp*^-/-^ MEFs (Fig. 7A and 7B). In our previous report, we demonstrated that CO induces TTP activation *via* Sirt1 [33]. To demonstrate the effects of Sirt1-dependent TTP activation on the migration of TTP into SGs, we used EX527 as a Sirt1 inhibitor. The increase of the migration of TTP into SGs by CORM2 was inhibited in EX527-treated AML-12 cells, which is due to the suppression of CO-induced TTP activation by EX527 (Fig. 7C). Next, we observed that CO-induced TTP activation facilitated the decrease of PAI-1 in ETO-treated primary MEFs (Fig. 7D and 7E) and AML-12 cells (Fig. 7F). In addition, EX527 inhibited the reduction of PAI-1 by CORM2 (Fig. 7D-7F). Taken together, our results demonstrate that CO-induced Sirt1-dependent TTP activation promoted TTP migration into SGs, leading to increased PAI-1 degradation.

## DISCUSSION

The study of aging is critical to overcoming diseases and maintaining life quality. In aged individuals, PAI-1 expression is elevated in a variety of pathologies associated with the aging process [36], including vascular sclerosis [41], cardiac and lung fibrosis [42], metabolic syndrome [43, 44], cancer [45], and inflammatory and stress responses [46].

In this study, we demonstrated that mice sustain age-dependent increases in PAI-1 expression. We also demonstrate that therapeutic application of CO promotes PAI-1 sequestration by SG assembly and reduced PAI-1 secretion *via* TTP activation.

**Figure 8.**
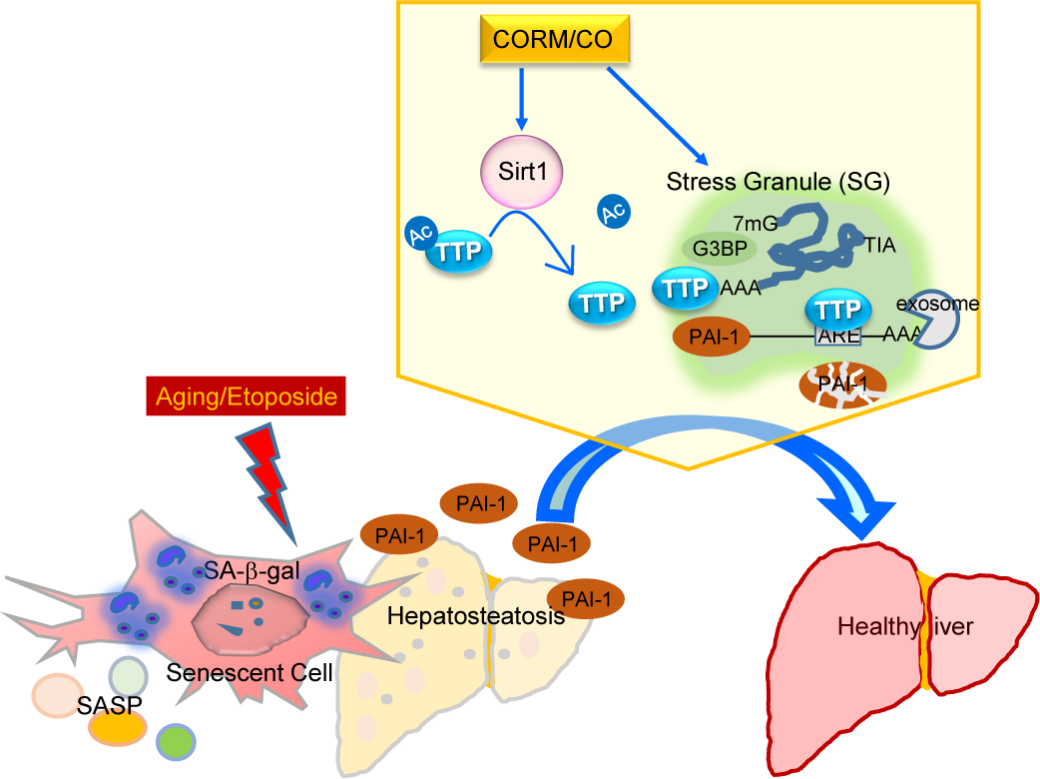
TTP can prevent liver injury and hepatocyte cell senescence during aging. TTP may protect against age-associated senescence phenotypes *via* decreasing PAI-1 levels. CO reversed ETO-induced cellular senescence *via* PAI-1 degradation, in cells expressing TTP. CO-induced SGs prevent cellular senescence by sequestering PAI-1. CO-induced Sirt1-dependent TTP activation promoted TTP migration into SGs, leading to increased PAI-1 degradation.

The hormesis effects of CO at low doses have been reported as the anti-inflammatory response [33], anti-obese effects [47, 48], and anti-oxidative response [49, 50]. CO, as a protector against cellular stress, activates the PERK-eIF2α signaling pathway through mtROS production, leading to induction of SG assembly [32]. The SG-mediated inhibition of senescence is caused by recruitment of PAI-1, a member of the SASP [30] and a well-known promoter of senescence [51-53]. Although CO has been shown to prevent bleomycin-induced cellular senescence by SG assembly [54], the underlying mechanisms remain unclear. In our previous reports, CO was shown to promote Sirt1 expression and activation, resulting in the deacetylation of p53 [55] and TTP [33]. Here, we demonstrate that the Sirt1 inhibitor prevents the CO-mediated migration of TTP into SGs. In contrast, phosphorylation of TTP induced by the p38-MAPK/MK2 pathway [56, 57] resulted in exclusion of TTP from SGs, leading to TTP:14-3-3 complex formation [29]. Given that SGs form from pools of untranslated mRNA and contain various translation initiation factors, as well as a variety of RNA-binding proteins and non-RNA-binding protein [58], Sirt1-mediated TTP deacetylation permitted TTP to bind to the ARE of PAI-1 mRNA in SGs. Therefore, TTP deacetylation by CO is critical to control aging. Aged *Ttp*^-/-^ mice showed higher PAI-1 levels than young *Ttp*^-/-^ mice or corresponding WT mice. Thus, the interaction between TTP and PAI-1 may play a critical role in aging.

Age-mediated NAFLD in *Ttp*^-/-^ mice represents the increase of inflammatory cytokines, SASP, and liver damage. Thus, we suggest that TTP activation can alleviate age-mediated NAFLD. Given that aging is characterized by cellular senescence, the role of TTP has been studied in cellular senescence. Cellular senescence is a state of stable proliferative arrest triggered by damaging signals such as DNA damage or oncogene-dependent pathways [23].

In this study, we treated human diploid fibroblast (WI-38) cells, and primary mouse embryonic fibroblasts MEFs, with ETO to establish DNA damage induced-cellular senescence. ETO, one of the topoisomerase II poisons, is commonly used as a chemotherapeutic agent, which can cause DNA double strand breaks (DSBs), which are toxic to normal cells [37]. Additionally, the treatment of normal human fibroblasts with ETO can lead to a long-term cell cycle arrest and premature senescence mediated by the activation of p53 and enhancement of CDK inhibitors, including p16^INK4a^ and p21^CIP1^ [59, 60]. We observed that CO exerts a strong anti-senescent effect on ETO-mediated premature senescence. Our results showed that the administration of CORM2 and exogenous CO gas can significantly decrease multiple hallmarks of cellular senescence, including the percentage of SA-β-gal positive cells, DNA damage associated γ-H2AX foci, CDK inhibitor p21 expression, and the expression of several SASPs, such as IL-6, TNF-α, and IL-1β. Consistent with *in vivo* aging results, TTP deficiency abolished the ability of CO to prevent ETO-mediated cellular senescence. Moreover, CO can cause the sequestration of PAI-1 into SGs during challenge with ETO, which can dramatically reduce the secretion of PAI-1. Notably, the reduction of PAI-1 by CO under these conditions was dependent on TTP activation. Further studies will be needed to validate these mechanisms in *in vivo* models. We conclude that CO-dependent TTP activation diminishes PAI-1 levels in SGs, leading to alleviation of age-dependent NAFLD and ETO-induced cellular senescence (Fig. 8). Therefore, we suggest that TTP activation by CO may represent a novel therapeutic strategy to ameliorate cellular senescence and aging-mediated diseases.

## Supplementary Materials

The Supplementary data can be found online at: www.aginganddisease.org/EN/10.14336/AD.2023.0120
