# Supplementary material for: CO-Induced TTP Activation Alleviates Cellular Senescence and Age-Dependent Hepatic Steatosis via Downregulation of PAI-1
Source: Aging Dis. 2023 Apr 1;14(2):484–501. doi: 10.14336/AD.2023.0120 (PMC10017156; doi:10.14336/AD.2023.0120)
Supplement: Supplementary file 1 — The Supplementary data can be found online at: www.aginganddisease.org/EN/10.14336/AD.2023.0120 [file AD-14-2-484-s.pdf]

SUPPLEMENTARY DATA

CO-Induced TTP Activation Alleviates Cellular Senescence and Age-Dependent Hepatic Steatosis via Downregulation of *PAI-1*

Jeongmin Park[1#], Yingqing Chen[2#], Jeongha Kim[3], Eunyeong Hwang[3], Gyu Hwan Park[4], Chae Ha Yang[3], Stefan W. Ryter[5], Jeong Woo Park[1], Hun Taeg Chung[1*], Yeonsoo Joe[1*]

© 2023. Park J et al. Published online at http://www.aginganddisease.org/EN/10.14336/AD.2023.0120

SUPPLEMENTARY DATA

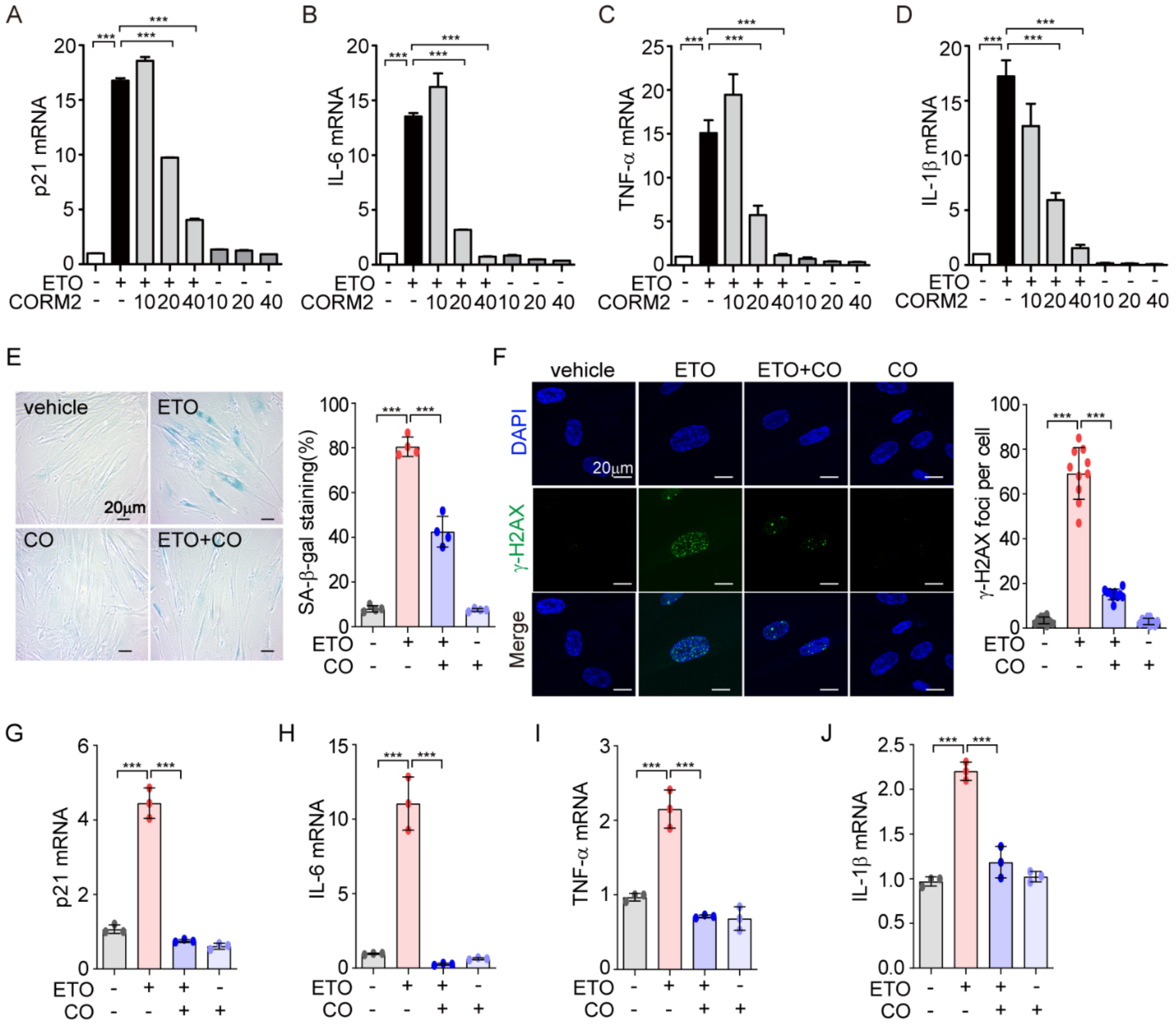

Supplementary Figure 1. CO inhibits ETO-induced cellular senescence in human and murine fibroblasts. (A-D) WI-38 cells were pre-treated with CORM2 at the indicated does (0, 10, 20, and 40 μM) for 6 h followed by the administration of ETO (80 μM) for 24 h and cells were refed with fresh media. During the process of senescence, cells were administrated with CORM2 for 6 h every two days, after 7 days treatment, cells were harvested and the mRNA expression of **(A)** p21, **(B)** IL-6, **(C)** TNF-α, and **(D)** IL-1β were analyzed by qRT-PCR. Data were analyzed using Kruskal-Wallis test followed by the Dunn post hoc test and expressed as means ± SD; *n*=3 biological replicates; $^{***}p<0.001$. **(E-J)** WI-38 cells were pretreated with exogenous CO gas (250 ppm) for 2 h, followed by the administration of ETO (80 μM) for 24 h and then cells were refed with fresh media. During the process of senescence, cells were post-treated with CO gas for 2 h every two days. After 7 days incubation, cells were performed with **(E)** SA-β-gal staining (mean ± SD; *n*=5 biological replicates; $^{***}p<0.001$; Kruskal-Wallis test followed by the Dunn post hoc test) and **(F)** immunofluorescence for detecting γ-H2AX foci (mean ± SD; *n*=10 biological replicates; $^{***}p<0.001$; one-way ANOVA followed by Tukey post hoc test). Mouse IgG1 was used as a negative control of anti-γ-H2AX antibody. The mRNA expression of **(G)** p21, **(H)** IL-6, **(I)** TNF-α, and **(J)** IL-1β were measured by qRT-PCR (mean ± SD; *n*=3 biological replicates; $^{***}p<0.001$; Kruskal-Wallis test followed by the Dunn post hoc test).

© 2023. Park J et al. Published online at http://www.aginganddisease.org/EN/10.14336/AD.2023.0120

SUPPLEMENTARY DATA

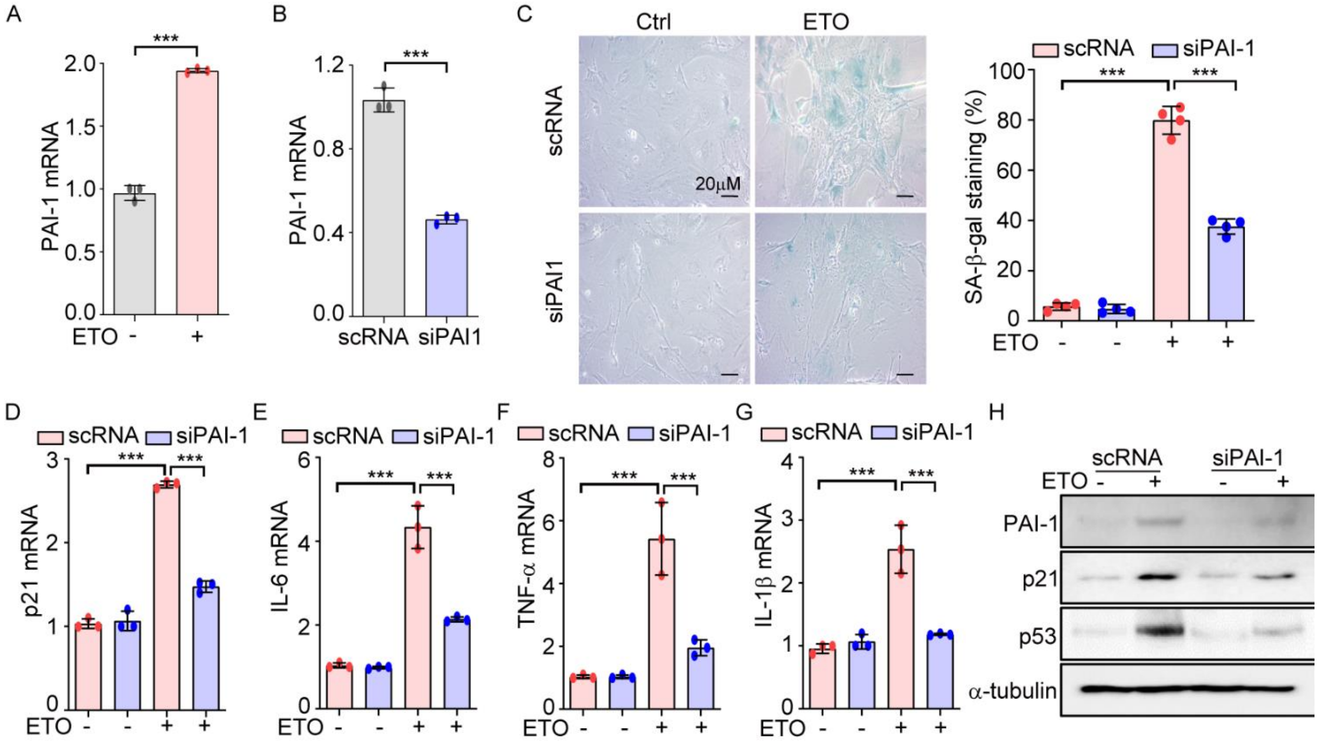

Supplementary Figure 2. PAI-1 is involved in ETO-induced premature senescence in WI-38 and MEF cells (A) Primary MEFs were treated with ETO (2 μM) for 4 days, and then cells were harvested, and the mRNA level of PAI-1 was measured by qRT-PCR (mean ± SD; *n*=3 biological replicates; $^{***}p<0.001$; Mann-Whitney U test). **(B-H)** Primary MEFs were transfected with scramble siRNA (scRNA) and siRNA against PAI-1 (siPAI-1) for 36 h, and then **(B)** PAI-1 mRNA level was determined by qRT-PCR (mean ± SD; *n*=3 biological replicates; $^{***}p<0.001$; Mann-Whitney U test). **(C)** Transfected cells were treated with etoposide (2 μM). After 4 days incubation, cells were subjected to SA-β-gal staining. Scale bar: 20 μm. Cells were analyzed to calculate the percentage of SA-β-gal-positive cells (mean ± SD; *n*=4 biological replicates; $^{***}p<0.001$; Kruskal-Wallis test followed by the Dunn post hoc test). The mRNA expression of **(D)** p21, **(E)** IL-6, **(F)** TNF-α, and **(G)** IL-1β were measured by qRT-PCR (mean ± SD; *n*=3 biological replicates; $^{***}p<0.001$; Kruskal-Wallis test followed by the Dunn post hoc test). **(H)** The protein expression of PAI-1, p21, and p53 were analyzed by immunoblotting.

© 2023. Park J et al. Published online at http://www.aginganddisease.org/EN/10.14336/AD.2023.0120

SUPPLEMENTARY DATA

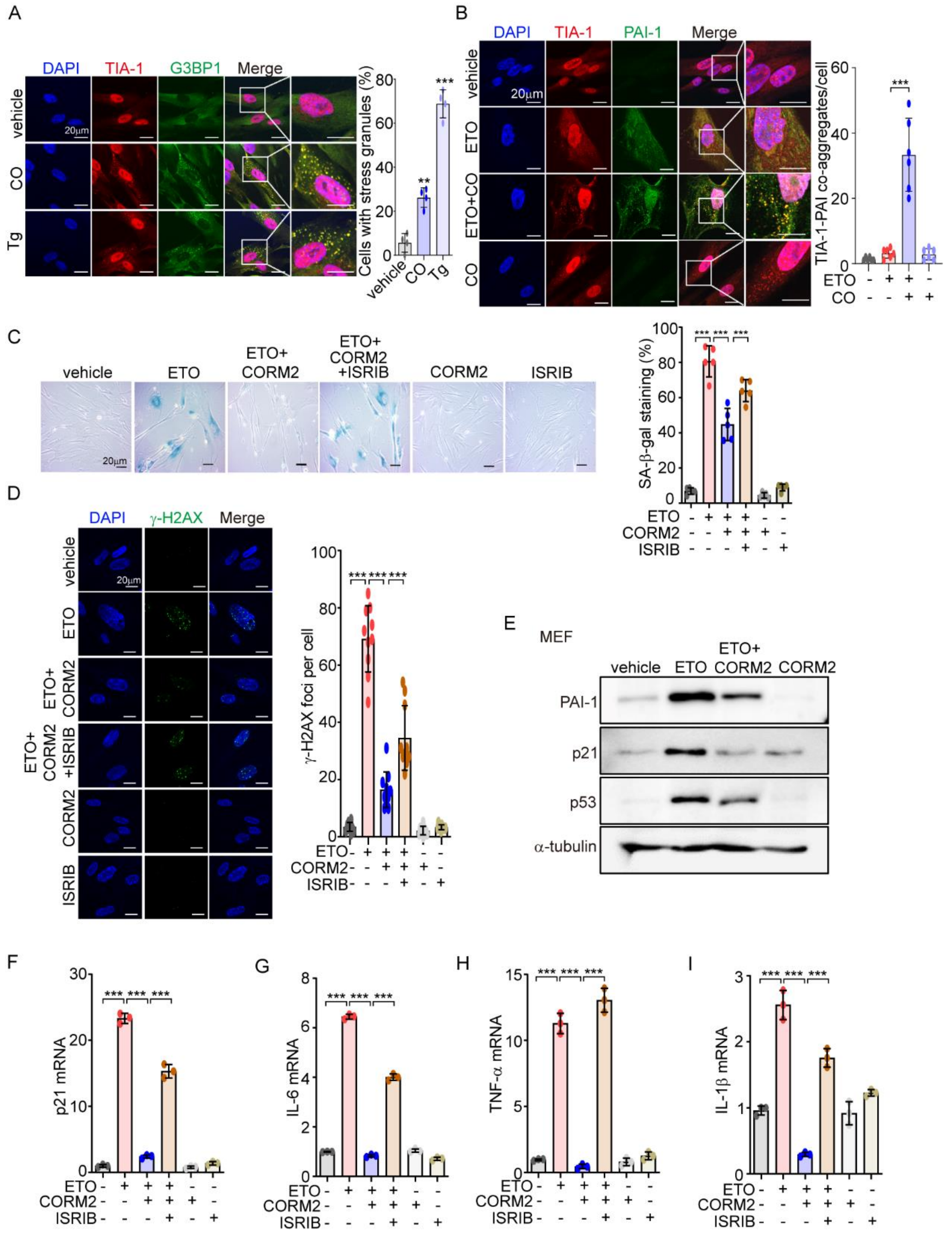

Supplementary Figure 3. CO-induced SGs participate in reducing ETO-induced senescence by sequestration of PAI-1 (A) WI-38 cells were treated with CO gas (250 ppm) for 6 h. 200 nM thapsigargin (Tg) was used as a positive control. An immunofluorescence assay was performed to detect the formation of SGs by visualizing the co-localization of TIA-1(red) and G3BP1 (green). Scale bar: 20 μm (*left*). The percentage of cells containing SGs was analyzed and is shown in the right panel (mean ± SD; *n*=4 biological replicates; $^{**}p<0.01$ and $^{***}p<0.001$; Kruskal-Wallis test followed by the Dunn post hoc test). **(B)** WI-38 cells were pretreated with CO gas (250 ppm) for 6 h followed by the administration of ETO (80 μM) for 24 h, and then cells were refed with fresh media. During the process of senescence, cells were post-treated with CO gas (250 ppm) for 6 h. After 7 days incubation, an immunofluorescence assay was performed to detect the TIA-1 (red) and PAI-1 (green) co-aggregates. Rabbit IgG and mouse IgG1 were used as a negative control of anti-TIA-1 antibody and anti-PAI-1 antibody, respectively. Scale bar: 20 μm (*left*). Quantification of co-localization of PAI-1 and TIA-

© 2023. Park J et al. Published online at http://www.aginganddisease.org/EN/10.14336/AD.2023.0120

1 is shown in the bar graphs to the right panel (mean ± SD; *n*=6 biological replicates; $^{***}p<0.001$; one-way ANOVA followed by Tukey post hoc test). **(C, D)** WI-38 cells were pretreated with CORM2 (40 μM) and ISRIB (200 nM) for 6 h followed by the treatment of ETO (80 μM) for 24 h, and then cells were refed with fresh media. During the process of senescence, cells were post-treated with CORM2 (40 μM) and ISRIB (200 nM) for 6 h. **(C)** After 7 days incubation, cells were fixed and stained for SA-β-gal. Scale bar: 20 μm (*left*). The percentage of SA-β-gal- positive cell was analyzed (*right*; mean ± SD; *n*=5 biological replicates; $^{***}p<0.001$; Kruskal-Wallis test followed by the Dunn post hoc test). **(D)** Cells were analyzed by immunofluorescence for γ-H2AX nuclear foci. Mouse IgG1 was used as a negative control of anti-γ-H2AX antibody. Scale bar: 20 μm (*left*). Quantification of γ-H2AX nuclear foci is shown in the bar graphs (*right*; mean ± SD; *n*=10 biological replicates; $^{***}p<0.001$; one-way ANOVA followed by Tukey post hoc test). **(E)** Levels of protein expression of PAI-1, p21, and p53 were detected by immunoblotting in the indicated groups. **(F-I)** The levels of mRNA expression of **(F)** p21, **(G)** IL-6, **(H)** TNF-α, and **(I)** IL-1β were detected by qRT-PCR in the indicated groups (mean ± SD; *n*=3 biological replicates; $^{***}p<0.001$; Kruskal-Wallis test followed by the Dunn post hoc test).

© 2023. Park J et al. Published online at http://www.aginganddisease.org/EN/10.14336/AD.2023.0120
